# Engineered Sensory Nerve Guides Self‐Adaptive Bone Healing via NGF‐TrkA Signaling Pathway

**DOI:** 10.1002/advs.202206155

**Published:** 2023-02-01

**Authors:** Zengjie Zhang, Fangqian Wang, Xin Huang, Hangxiang Sun, Jianxiang Xu, Hao Qu, Xiaobo Yan, Wei Shi, Wangsiyuan Teng, Xiaoqiang Jin, Zhenxuan Shao, Yongxing Zhang, Shenzhi Zhao, Yan Wu, Zhaoming Ye, Xiaohua Yu

**Affiliations:** ^1^ Department of Orthopedic Surgery The Second Affiliated Hospital Zhejiang University School of Medicine Orthopedics Research Institute of Zhejiang University Key Laboratory of Motor System Disease Research and Precision Therapy of Zhejiang Province 88 Jiefang Road Hangzhou City Zhejiang Province 310003 P. R. China; ^2^ Department of Orthopedic Taizhou First People's Hospital Wenzhou Medical University 218 Hengjie Road, Huangyan District Taizhou City Zhejiang Province 318020 P. R. China

**Keywords:** BMP‐2, extracellular matrix, nerve growth factor, osteogenesis, sensory nerve

## Abstract

The upstream role of sensory innervation during bone homeostasis is widely underestimated in bone repairing strategies. Herein, a neuromodulation approach is proposed to orchestrate bone defect healing by constructing engineered sensory nerves (eSN) in situ to leverage the adaptation feature of SN during tissue formation. NGF liberated from ECM‐constructed eSN effectively promotes sensory neuron differentiation and enhances CGRP secretion, which lead to improved RAOECs mobility and osteogenic differentiation of BMSC. In turn, such eSN effectively drives ossification in vivo via NGF‐TrkA signaling pathway, which substantially accelerates critical size bone defect healing. More importantly, eSN also adaptively suppresses excessive bone formation and promotes bone remodeling by activating osteoclasts via CGRP‐dependent mechanism when combined with BMP‐2 delivery, which ingeniously alleviates side effects of BMP‐2. In sum, this eSN approach offers a valuable avenue to harness the adaptive role of neural system to optimize bone homeostasis under various clinical scenario.

## Graphical abstract

The adaptation feature of sensory nerve during tissue formation is leveraged to propose a neuromodulation approach to orchestrate bone defect healing by constructing engineered sensory nerves (eSN) in situ. eSN sustained‐release NGF to promote sensory nerve reinnervation, which regulates MSC osteogenic differentiation, vascular regeneration, and regulates osteoblast and osteoclasts to participate in bone remodeling and guides self‐adaptive bone healing.

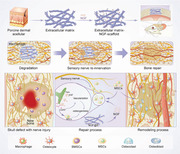

## 1 Introduction

Critical size bone defect caused by trauma and other diseases presents a challenging clinical scenario with no satisfactory therapeutic options.^[^ ^1^ ^]^ Such challenge is rooted in the fact that bone repair is a complex multi‐stage process involving inflammation, neurovascular network reconstruction, rapid bone mineralization, and bone remodeling.^[^ ^2^ ^]^ In response, bone healing strategies targeting at one or more stages of this process have been developed to accelerate bone formation. For instance, dual VEGF/BMP‐2 delivery systems were designed to simultaneously promote angiogenesis and osteogenesis in order to achieve vascularized bone healing.^[^ ^3^ ^]^ More recently, immunomodulatory biomaterials have developed to leverage the anti‐inflammatory property of immune cells such as macrophages to facilitate bone formation.^[^ ^4^ ^]^ However, the role of nerve system has been largely underestimated in the context of bone regeneration. In fact, bone is well‐innervated with both sensory and autonomic nerve fibers, which distribute through periosteum, bone marrow, growth plate, and mineralized trabecular/cortical bone. Accumulating evidences indicate that neural system plays irreplaceable role in skeleton development and metabolism by directly or indirectly regulating the activities of osteoblasts and osteoclasts,^[^ ^5^ ^]^ thus it is conceivable to develop novel bone healing strategy harnessing regulatory function of nerves during bone healing.

The multiple regulatory roles of sensory nerves during bone metabolism have been gradually identified besides its function as signal transduction systems.^[^ ^6^ ^]^ Clinical studies and animal researches demonstrated that the loss of sensory nerves prone to increased bone loss and delayed fracture healing.^[^ ^7^ ^]^ Toru Fukuda et al. first demonstrated the relationship between sensory nerve innervation and bone metabolism.^[^ ^8^ ^]^ They found that the deleting the axon‐guiding factor‐Sema3A in neurons leads to the decreased sensory nerve innervation in bones, resulting in an imbalance of osteoblast and osteoclast, which accelerated osteopenia.^[^ ^9^ ^]^ Chen et al. reported that sensory nerve may sense the signaling of bone metabolic activity by PGE2 receptor 4(EP4) to regulate mesenchymal stromal cell lineage differentiation.^[^ ^10^ ^]^ In addition, sensory nerves are known to drive the subsequent reconstruction of vascular network which are responsible for the ability to regenerate entire bone tissues. Zhang et al. reported that secreted calcitonin gene‐related peptide (CGRP) by sensory neurons positively regulate vascular network reconstruction.^[^ ^11^ ^]^ Moreover, sensory nerve secreted substance P (SP), CGRP, and other neuropeptides to modulate proliferation and osteogenic differentiation of MSCs.^[^ ^12^ ^]^ Due to the versatile roles of sensory nerves in bone metabolism and homeostasis, reconstruction of sensory nerve network at the bone defect sites may be considered as a feasible alternative to regulate new bone formation.

Tissue development dictates corresponding innervation processes including its type and density, which are controlled by activating distinct tyrosine kinase receptors via secreting neurotrophies. Current data suggest that higher expression of nerve growth factor (NGF) was observed in bone fracture and NGF activate its receptor TrkA to determine the type and density of invading nerves during bone healing.^[^ ^13^ ^]^ Tomlinson et al. demonstrated that locoregional deletion of NGF in monocyte/macrophage or inactivation of TrkA signaling impaired sensory nerve innervation, blunted vascularization, and delayed formation of primary and secondary ossification centers.^[^ ^13^, ^14^ ^]^ In addition, James et al. found that temporal inhibition of TrkA catalytic activity resulted in similar deficiencies in nerve regrowth, revascularization, and fracture healing.^[^ ^15^ ^]^ Thus, NGF‐TrkA signaling pathway may be leveraged to construct neuro‐modulatory approaches to promote bone regeneration by providing an innervated niche for sensory nerve guided ossification.

**Scheme 1:**
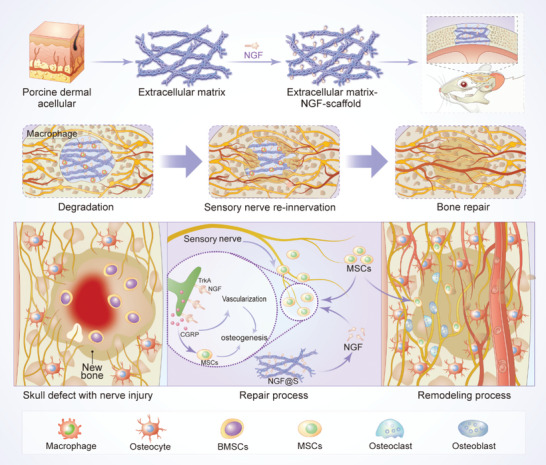
Engineered sensory nerve guides self‐adaptive bone healing via NGF‐TrkA signaling pathway: The adsorption capacity of the acellular scaffold was leveraged to construct a sustained release system of NGF (NGF@S), which promoted the sensory nerve reinnervation at the site of bone tissue injury, and then promoted bone repair. Engineered sensory nerve system sustained‐release NGF to promote sensory nerve reinnervation through NGF‐TrkA signaling pathway and reinnervated sensory nerve secretes CGRP to promote MSC osteogenic differentiation and vascular regeneration in tissues. In addition, sensory nerve regulates osteoblast and osteoclasts to participate in bone remodeling and guides self‐adaptive bone healing.

In this work, we aimed to construct an engineered neurotrophic microenvironment by non‐covalently incorporating NGF into ECM derived 3D scaffolds to achieve engineered sensory nerve‐mediated bone regeneration (**Scheme** **1**). In particular, our objective was spirited into two folds: 1) Whether engineered sensory nerves induced by sustained NGF release could play as a mediator for ossification center formation via NGF‐TrkA signaling pathway? 2) Whether engineered sensory nerve demonstrate an adaptive response to other regulatory factors such as BMP‐2? To realize such purposes, NGF‐TrkA signaling pathway was leveraged to realize sensory innervation and further promote innervated bone regeneration via a local NGF releasing scaffold where NGF is non‐covalently bound on scaffolds through its intrinsic affinity to ECM. The biological effects of engineered sensory nerve on vascularization and osteogenic differentiation of mesenchymal stem cells were evaluated using multiple cell culture models. Next, both subcutaneous and calvarial bone defect models demonstrated engineered sensory nerve effectively drove ossification under the guidance of engineered sensory nerve. Finally, we also found that engineered sensory nerve was able to adaptively suppress excessive bone formation and promote bone remodeling by activating osteoclasts via CGRP‐dependent mechanism when combined with BMP‐2 delivery.

## 2 Results and Discussion

### 2.1 Active Bone Formation Is Highly Co‐Localized with NGF‐Mediated Sensory Nerve Innervation in Calvarial Defect

**Figure 1:**
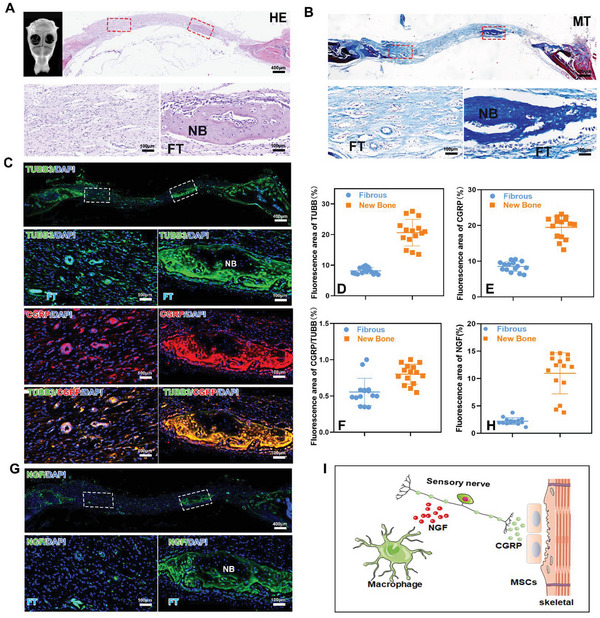
Active bone formation is highly co‐localized with NGF‐mediated sensory nerve innervation. A,B) Representative tile scans of H&E‐stained images and Masson‐stained images of cranial defect. C,G) IHC was performed on a fracture callus on day 3 after injury, including staining for TUBB, CGRP, and NGF; Semiquantitative analysis of D) TUBB3, E) CGRP, F) CGRP/TUBB3, and H) NGF expression on day 15 after cranial defect. I) Schematic of hypothesis that macrophage secreted NGF to promote sensory nerve re‐innervation, which in turn secrete CGRP to regulate bone regeneration. *n* = 3 animals per group. Data are represented as mean ± SD.

Although the role of sensory nerve in bone homeostasis has been reported, its spatiotemporal occurrence and distribution during bone defect healing remains vague. We performed immunohistochemical staining of pan‐neuronal marker TUBB3 and sensory nerve marker CGRP to compare the distribution of peripheral nerves in the injured and uninjured skull. Besides the normal skull anatomy shown in HE and Masson staining, TUBB3‐positive nerve fibers were mainly observed parallel to surface of the cranial and a few travels through the bone marrow foramen. It is noteworthy that the majority of the nerve fibers found in uninjured skull were CGRP‐positive (Figure S1, Supporting Information). In contrast, TUBB‐positive and CGRP‐positive nerve fibers were found to highly co‐localize with new bone callus, but rarely observed near poster frontal suture (**Figure** **1**C–F), which is consistent with previous report that NGF reporter activity was abundant around newborn bone tissue, especially around CGRP‐positive nerve fibers on the area of the cranial defect^[^ ^14^, ^15^ ^]^ (Figure 1G,H). These results indicates that new bone formation was highly correlated with sensory nerve re‐innervation (Figure 1I).

### 2.2 Neural Inductive NGF@S Construction and Its Regulatory Effects on Osteogenesis and Angiogenesis In Vitro

**Figure 2:**
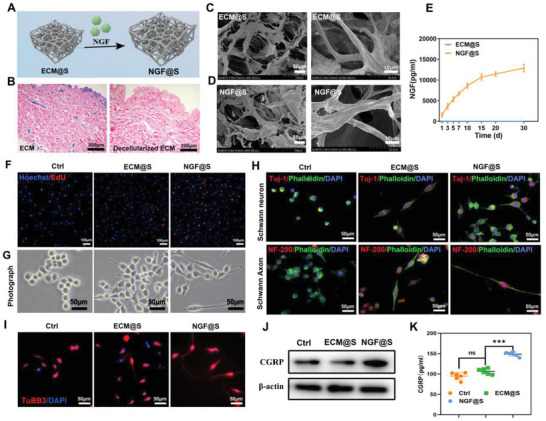
The construction of NGF@S and their effects for nerve regeneration in vitro. A) Schematic illustration of the construction of the NGF@S scaffold; B) HE‐stained images of ECM and decellularized ECM; C,D) Representative scanning electron microscopy (SEM) of ECM@S and NGF@S; E) NGF release curve of NGF@S in SBF; F) Cell proliferation of Schwann cells co‐cultured with different scaffolds were evaluated by Edu kit. G) Immunofluorescence staining of Tuj‐1 and NF‐200 images on Schwann cells after co‐culture with different scaffolds. I) Immunofluorescence staining of TUBB3 images on Dorsal root ganglion cells after co‐culture with different scaffolds. J,K) CGRP expression and CGRP secretion of Dorsal root ganglion cells with different scaffolds were detected respectively by WB and ELISA. *n* = 3 times per experiments. Data are represented as mean ± SD.

Since we confirmed the essential role of NGF during sensory nerve mediated bone formation, we next constructed sensory nerve‐inductive scaffolds (NGF@S) by binding NGF onto porcine dermis derived extracellular matrix nanofibrous scaffolds via the high affinity between NGF and ECM (**Figure** **2**A). After confirming the complete removal of cellular components in porcine dermis (Figure 2B), NGF@S was fabricated by freeze‐drying to form porous structure between the interlacing collagen fibers (Figure 2C). Besides, proteomics analysis demonstrated that the acellular materials were mainly composed of extracellular matrix collagen and adhesive proteins (pink), accounting for 92.04% (Figure S2, Supporting Information). The natural porous structure of ECM and multiple growth factor binding sites provided by collagen and ECM glycoproteins were leveraged to realize spatiotemporal controlled loading and release of NGF.^[^ ^16^ ^]^ SEM of NGF@S performed that binding NGF to ECM maintained the original fiber staggered microstructure (Figure 2C,D) and sustained release of NGF was maintained for more than 30 days (Figure 2E).

Next, the ability of NGF@S to promote differentiation and neurite growth of Schwann cells and rat dorsal root ganglion cells (DRGs) in vitro, as well as the nerve‐bone crosstalk were evaluated via Edu positive staining and immunofluorescence. As shown in Figure 2F,G, Edu positive cells and elongated Schwann cells substantially increased in NGF@S group when compared with control and ECM@S group. Immunofluorescence staining showed that the morphology of Schwann cells cultured with NGF@S changed from spherical to fusiform, axons lengthened, and the distribution of neural differentiation factors Tuj‐1 and NF‐200 increased compared with control and ECM group (Figure 2H). Similar results of significant axonal lengthening of rat DRGs were observed with the co‐culture with NGF@S (Figure 2I). Since sensory nerves secret several neurotransmitters such as CGRP and CGRP is considered as an important regulator of bone healing process, it is suggested that nerves may regulate bone repair process by secreting CGRP.^[^ ^17^ ^]^ We further investigate the effect of NGF@S on the expression and secretion of CGRP in DRGs using a co‐culture model. As shown in Figure 2J, western blots shown that sustained release of NGF from NGF@S promoted the expression of CGRP in DRGs. In addition, the concentration of CGRP in NGF@S culture medium (150 pg mL^−1^, Figure 2K) was significantly higher than ECM@S group and control group, indicating that NGF promoted the secretion of CGRP, which may serve as driving force to promote tissue healing.

**Figure 3:**
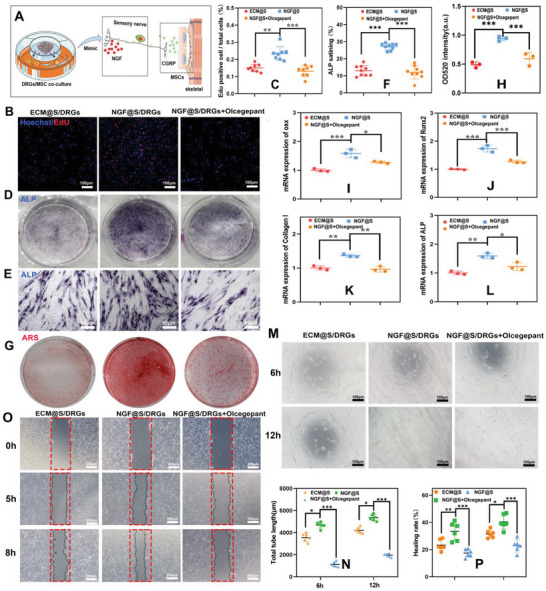
DRGs secret CGRP to regulate BMSC osteogenic differentiation co‐cultured with NGF@S. A) Schematic illustration of DRGs/scaffolds co‐culture with BMSCs. BMSCs or RAOECs were co‐cultured with DRGs and different scaffolds with or without Olcegepant. B) The proliferation of BMSCs with DRGs/Scaffold system were evaluated by Edu kit. C) Quantification analysis of Edu evaluation. D,E) ALP staining images of BMSCs with DRGs/scaffold system after 7 days co‐culture. F) Quantification analysis of ALP staining. G,H) ARS staining images of BMSCs with DRGs/scaffold system after 14 days and quantification analysis of ARS staining. Osteogenic differentiation related genes mRNA expression of I) OSX, J) RUNX‐2, K) Collagen I, L) ALP, with DRGs/scaffold system after 14 days. M) Representative optical microscopic images of RAOECs tube formation assay at 6 h and 12 h, respectively. N) Quantification analysis of RAOECs tube formation. O) Representative optical microscopic images of scratch line at 0, 5, and 8 h, respectively. P) Quantification analysis of scratch test. *n* = 3 times per experiments. Data are represented as mean ± SD.

To validate whether NGF@S regulated rat BMSCs osteogenic differentiation through CGRP secreted by regenerated sensory nerves, a BMSC/DRGs/scaffold co‐culture model was constructed in vitro (**Figure** **3**A). We detected an increase in Edu positive cells in the DRGs co‐cultured group, whereas a significant decrease in the number of Edu positive cells after the addition of CGRP inhibitors (Olcegepant, Olc) (Figure 3B,C). In addition to promoting proliferation, we further investigated the effect of NGF@S/DRGs in BMSCs differentiation by Alkaline phosphatase (ALP) and Alizarin red (ARS) staining. ALP results shown that deeper staining and more positive BMSCs in the NGF@S group, but the olcegepant could counteract this effect (Figure 3D–F). Similar to ALP staining, ARS staining showed that more mineralized nodules were observed in NGF@S group comparison to control group, while inhibition of CGRP significantly decreased the number of mineralized nodules (Figure 3G,H). Moreover, NGF@S promoted the expression of osteogenesis differentiation related mRNA such as *Runx‐2, Collagen I, Alp*, and *Osx*, whereas olcegepant reversed this phenomenon (Figure 3I–L). Previous literatures demonstrated that NGF derived from surrounding infiltrated monocytes activate sensory nerve via TrkA receptor to promote the accumulation of CGRP vesicles and exocytosis. The sensory nerve released CGRP, in turn, promoted osteogenic differentiation via CGRP‐cAMP‐CREB signaling pathway.^[^ ^15^ ^]^ These results indicate that NGF@S promoted the proliferation and differentiation of nerve cells, which in turn promoted CGRP‐mediated osteogenic differentiation of BMSC.

Vascularization is tightly associated with bone regeneration, thus impact of innervation on vascularization during bone healing was assessed as nerve fibers and blood vessels are usually accompanied with each other and mutual signals affect their growth and activity. Previous study showed that CGRP secreted by neural cells contributed to bone healing via enhanced vascularization apart from direct regulating the osteogenic differentiation of BMSCs. As such, we next validated whether sensory nerve promoted vascularization through secretion of CGRP by evaluating the tube formation and healing of hum Rat Aortic endothelial cell (RAOECs) in DRGs and RAOECs co‐culture system. As shown in Figure 3M, RAOECs incubated with NGF@S and DRGs significantly increased the tubule length in comparison with ECM@S/DRGs group, but the tubule length was significantly decreased when CGRP inhibitor added to NGF@S/DRGs co‐culture system (Figure 3M,N). In addition, higher gap healing rates were observed in NGF@S/DRGs group (33% increase) compared to ECM@S group 5 h after scratch. When olcegepant was added to condition medium, gap healing rates were obviously decreased (51% decrease). Similar results were observed 8 h after scratch (Figure 3O,P), indicating that paracrine CGRP secreted by engineered sensory nerves was able to promote angiogenesis, which may synergistically contribute to vascularized bone regeneration.

### 2.3 Subcutaneous Implantation of NGF@S Induced Osteogenesis via Enhanced Sensory Nerve Innervation

**Figure 4:**
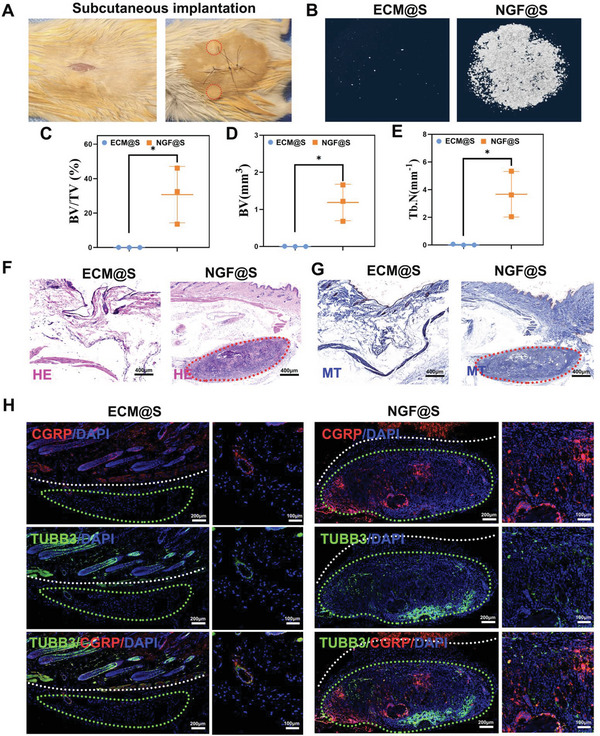
NGF@S promoted scaffold mineralization under subcutaneous. A) Photograph of a scaffold subcutaneous implantation experiment in Balb/c mice. B) 3D reconstruction of micro‐CT images with multiple metrics of µCT quantitative including C) BV, D) BV/TV, E) TB/N. F,G) HE‐stained and Masson‐stained images of subcutaneous tissue in different groups (scale bar:400 µm，Red circle：implaned materials). H) TUBB3 and CGRP immunofluorescence staining of subcutaneous tissues on 30 days after implantation (scale bar: 200 µm or 100 µm). *n* = 3 animals per time point and per group, each animal was embedded with two scaffolds. Data are represented as mean ± SD.

The ability of the NGF@S constructs to induce sensory nerve innervation osteogenesis was further evaluated in a Balb/c mouse subcutaneous implantation model (**Figure** **4**A). Both NGF@S and ECM scaffold were implanted subcutaneously for 4 weeks to evaluate new bone and nerve network formation surrounding the scaffolds. µCT observation showed no ossification activity took place in ECM group while obvious mineralized tissue was detected in NGF@S group (Figure 4B). Multiple metrics of µCT quantitative including BV, BV/TV, TB/N were significantly increased in NGF@S after 1‐month subcutaneous implantation. Consistent with µCT results, HE staining and Masson staining confirmed a significant increase of ossification level in NGF@S, while scaffolds in the control group were almost completely degraded without bone tissue formation. To evaluate the re‐innervation potential of NGF@S, immunofluorescent staining of TUBB3 and CGRP were performed. As shown in Figure 4H, TUBB3 positive nerve fibers were again predominantly at site of implantation. A dramatic increase in TUBB3 positive nerve fibers was observed in neuromodulation scaffold group, while almost no nerve fibers are detected in the implantation site in ECM group. In addition, an increase in CGRP fluorescence intensity was found in NGF@S group comparison to control group, and CGRP and TUBB fluorescence is highly co‐localized, indicating majority regenerated nerve fibers was sensory nerve (Figure 4H). These results suggest that NGF‐mediated sensory nerve innervation may be capable of inducing ossification within implants.

### 2.4 NGF@S‐Mediated Engineered Sensory Nerves Promoted Critical Size Bone Defect Healing

**Figure 5:**
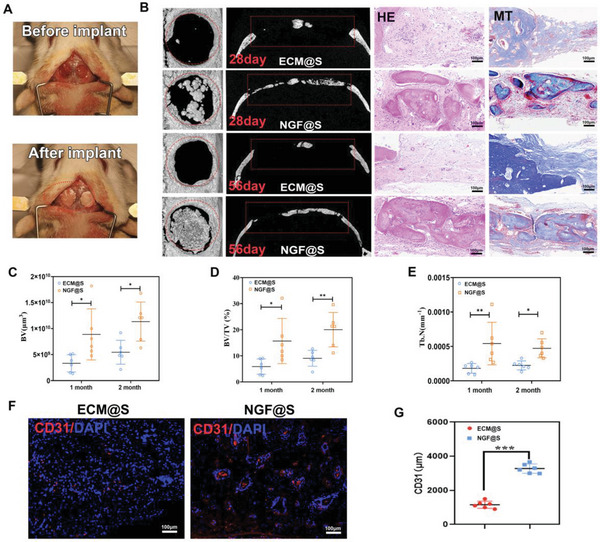
NGF@S promoted critical size bone defect healing. A) Photograph of a scaffold cranial defect implantation experiment in rats. B) HE‐stained and Masson‐stained images of cranial defect in different groups at 28 days or 56 days after implantation (scale bar: 100 µm), and 3D reconstruction of micro‐CT images with multiple metrics of µCT quantitative including C) BV, D) BV/TV, E) TB/N. F) CD31 immunofluorescence staining of cranial defect in G) different groups and quantified; *n* = 3 animals with 6 bone defects per time point and per group. Data are represented as mean ± SD.

Although NGF@S medicated innervation enhanced ectopic ossification subcutaneously, its influence to bone defects under bony condition remained unknown. Next, rat calvarial defect model was employed to assess the impact of engineered sensory nerve innervation on bone defect healing (**Figure** **5**A). 3D reconstruction of µCT showed that large area of new bone formed in NGF@S group while the defect in ECM group remined empty at day 28 (Figure 5B; Figure S3, Supporting Information). More defect area was covered by new bone in NGF@S group at day 56. HE and Masson Staining showed that more bone formed in NGF@S group. Consistent with this observation, quantitative µCT metrics of bone healing was increased in NGF@S group, including BV (Figure 5C, 57.5% increase), BV/TV (Figure 5D, 62.5% increase), Tb/N (Figure 5E, 66.4% increase) 28 days post‐surgery. Similar trends were observed after 56 days post‐surgery. We next evaluated vascularization effect of NGF@S using CD31 immunofluorescence staining since there is cross‐talk between bone regeneration, nerve re‐innervation, and angiogenesis. As shown in Figure 5G, both intensity and distribution of CD31 fluorescence signal were stronger in NGF@S group (3500 µm per section) than ECM@S scaffold (2000 µm per section), indicating that reinnervation mediated by NGF promoted local vascular growth within the defect (Figure 5G,H). Together, these data suggest that sustained release of NGF@S induced engineered sensory nerve innervation and effectively promoted new bone formation and vascularization.

**Figure 6:**
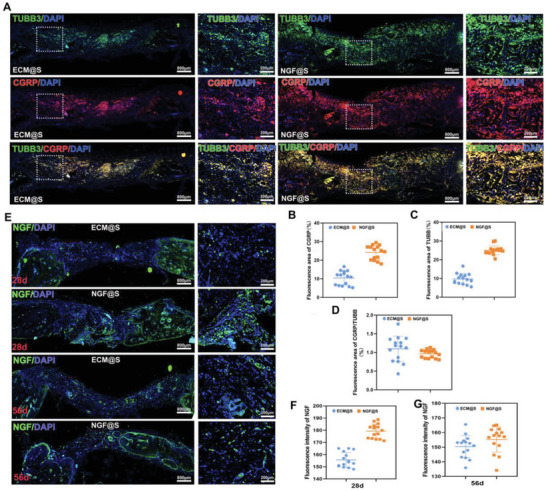
NGF@S promoted critical size bone defect healing through sensory nerve reinnervation. A) Representative fluorescence images showing TUBB3^+^ nerve fibers (Green) and CGRP^+^ nerve fibers (Red) in the cranial bone after scaffold implantation for 15 days. Semiquantitative analysis of B) the fluorescence distribution CGRP, C) TUBB3, and D) ratio of CGRP/TUBB. E) Representative fluorescence of NGF (Green) and nucleus (DAPI, Blue) in cranial bone after scaffold implantation for 28 or 56 days and F,G) quantified. *n* = 3 animals with 6 bone defects per time point and per group. Data are represented as mean ± SD.

Although bone defect healing assessment suggests high correlation between bone formation and NGF@S, the relationship between engineered sensory nerve and bone formation at molecular level has not been established. The domains of calvarium‐associated nerve fibers were next characterized using TUBB3 and CGRP immunohistochemical staining at 15 days after bone injury. Scanning of defect area showed that nerve fibers were most abundant at the leading bone edges of the healing defect, suggesting engineered sensory nerves are closely associated with newly formed bone. Moreover, majority of the TUBB‐positive nerve fibers were heavily overlapped with CGRP‐labeled nerve fibers (**Figure** **6**B). Quantitative analysis of fluorescence showed an increased intensity of CGRP (Figure 6B, 56.88%, increase) and TUBB3 (Figure 6C, 67.3%, increase) in NGF@S compared with ECM@S group. Consistent with fluorescence image, the fluorescence intensity ratio of CGRP/TUBB was close to 1 in each group, suggesting that most of the sensory nerve fibers induced by NGF was co‐located with new bone (Figure 6A–D). Distribution of NGF was also stained at the defect sites. Interestingly, large amount of NGF reporter was detected in the bone defect area in NGF@S group at day 28 while such high NGF expression substantially decreased at day 56 (Figure 6E,F,G). In line with previous reports, NGF reporter peaked in the early stages of bone healing, then gradually returned to normal expression levels, which suggests that bone regeneration was accompanied by reinnervation of sensory nerves.

These results demonstrate that construction of engineered sensory nerve may be considered as a valuable complement to current bone healing portfolio. Although various bone regeneration strategies leveraging different physiological characteristics including vascularization, immunity, etc. have been developed, the essential role of innervation has rarely been considered in the context of bone healing. Large body of literature supports an evolutionarily conserved role of sensory nerve in organogenesis and tissue regeneration.^[^ ^18^ ^]^ Toru et, al. reported that mice lacking Sema3a in neurons resulted in abnormal neuronal innervations, leading to a decreased bone formation.^[^ ^8^ ^]^ Temporal inhibition of TrkA signaling in TrkA F592A mice impaired nerve‐innervation, thereby delayed secondary ossification centers, decreased numbers of Osx‐expressing osteoprogenitors, and decreased femoral length and volume.^[^ ^15^ ^]^ By reinnervating the bone defects with engineered sensory nerves via NGF delivery system, we recapitulated innervated bone formation, which also activated multiple key processes including angiogenesis and osteogenesis during bone regeneration (Figures 5 and 6). Furthermore, it has been extensively reported that the nerve system is capable of providing adaptive response to external stimuli such as mechanical loading, we then wonder whether the engineered sensory nerve approach could regulate bone homeostasis in self‐adaption mode. In other words, could engineered sensory nerve play the role of “brain” and offer adaptive control over bone metabolism during bone healing?

### 2.5 NGF@S Optimized BMP‐2 Induced Bone Healing by Inhibiting Excess Bone Formation

**Figure 7:**
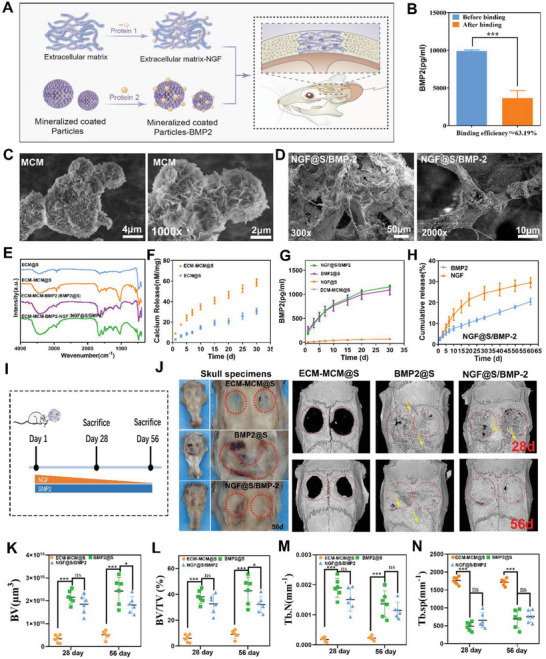
NGF@S optimized BMP‐2 induced bone healing. A) Schematic illustration of the construction of NGF and BMP2 sequential sustained release system (NGF@S/BMP‐2). B) Binding efficiency of mineralized coated microparticles (MCM) with BMP2. C,D) Representative Scanning electron microscope image for MCM and NGF@S/BMP‐2. White scale bar as shown in picture. The characteristic of constructed system was measured, including E) FITR, F) calcium release, G) BMP‐2 release curve, and H) BMP2 and NGF release cumulative release curve. *n* = 3 times experiments. Data are represented as mean ± SD. I) Schematic illustration of animal experiments. J) Representative photograph and reconstruction µCT images of cranial bone in each group at 28‐ or 56‐days post‐surgery. The bone volume (BV), bone volume fraction (BV/TV), trabecular number (Tb.N), and trabecular separation (Tb.sp) of the different groups. *n* = 3 animals with 6 bone defects per time point and per group. Data are represented as mean ± SD.

Both experimental and clinical evidence have demonstrated autonomic nervous system may involvement in bone remodeling, and disturbance of the autonomic nervous system could induce abnormal bone remodeling.^[^ ^19^ ^]^ Orthopedic application of BMP‐2 suffers from uncontrolled excessive bone formation,^[^ ^20^ ^]^ which might be due to the lack of neural regulation during bone healing. We hypothesized that BMP‐2 mediated excessive osteogenesis might be alleviated via reinnervation via engineered sensory nerve. To test this hypothesis, we constructed a BMP‐2/NGF dual release system to investigate the crosstalk between BMP‐2 mediated osteogenesis and NGF‐engineered sensory nerve (**Figure** **7**A,B; Figure S4, Supporting Information). Characteristics of the constructed scaffold (NGF@S/BMP‐2) were presented in Figure 7B–H and Figure S5, Supporting Information. BMP‐2 was immobilized on mineral coated microparticle according to our previous study and NGF was successfully encapsulated in ECM scaffold, which together achieved sequential release NGF and BMP2. Specifically, NGF bound on ECM was released in a relatively rapid rate in the first 15 days and peaks around 20 days, while BMP‐2 releases exhibited a more sustained release pattern (Figure 7G,H). In addition, the live and dead assay results and SEM images shown that modified Scaffold possess excellent biocompatibility (Figure S6, Supporting Information). With the advantages of sustained release and protein bioactivity preservation of mineral particles,^[^ ^21^ ^]^ this system might serve well to study the synergistic relationship between engineered sensory nerve and osteogenesis (Figure 7).

To investigate whether engineered sensory nerve could adaptively regulate BMP‐2 mediated bone formation, we evaluated the bone healing process at different time points (Figure 7I). While control group showed no healing sign at day 28, large amount of new bone was found to cover the cranial defect site, as well as the surrounding area in both BMP‐2@S and NGF@S/BMP‐2 groups (Figure 7J, the range marked in orange). µCT reconstruction also confirmed that ectopic osteogenesis was found in both single growth factor and dual factor groups (yellow arrow) at 28‐day post‐operation. However, a large area of hyperplastic new bone was observed only in BMP‐2@S group as this new bone completely covered the sagittal suture and coronal suture at 58‐day post‐operation. As for BMP‐2/NGF@S group, although some ectopic bone forms and covers the coronal suture at 28 days, no ectopic bone was found to cover the defect site at 56 days, indicating active bone remodeling in BMP‐2/NGF@S group took place at late stage of bone healing (Figure 7J). Quantitative µCT analysis further verified the above observations: BV and BV/TV of NGF@S/BMP‐2 group were similar to BMP‐2@S at day 28, however these values were significantly lower than BMP‐2@S at day 56, suggesting robust bone remodeling activity took place in NGF@S/BMP‐2 group. H&E and Masson staining also found that excessive ossification was inhibited in NGF@S/BMP‐2 group (Figure S7, Supporting Information).

**Figure 8:**
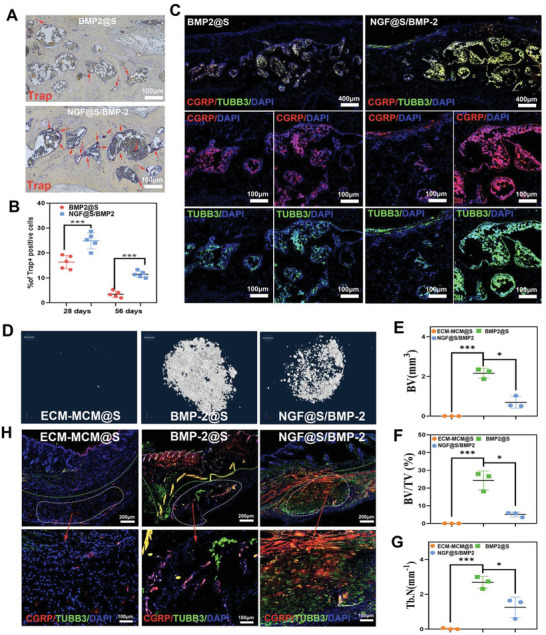
NGF@S optimized BMP‐2 induced bone healing were associated with sensory nerve re‐innervation. A) Representative images of trap staining of cranial bone in different groups and B) quantification of trap positive cells in each group. C) Representative fluorescence images showing TUBB3^+^ nerve fibers (Green) and CGRP^+^ nerve fibers (Red) in the cranial bone after scaffold implantation for 30 days. D) 3D reconstruction of micro‐CT images of scaffold implanted subcutaneous with multiple metrics of µCT quantitative including E) BV, F) BV/TV, G) TB/N. H) TUBB3 and CGRP immunofluorescence staining of subcutaneous tissues on 30 days after implantation. *n* = 3 animals per group. Data are represented as mean ± SD.

Since we observed excessive bone formation was substantially inhibited in the presence of NGF‐induced engineered sensory nerve,^[^ ^22^ ^]^ we next tried to dissect the regulatory role of sensory nerve by investigating the balance shift of bone metabolism during bone healing.^[^ ^23^ ^]^ We characterized the osteoclast activities at defect sites via TRAP staining to evaluate the remodeling phase of bone repair. On day 28, there were a large amount of TRAP^+^ multinucleated cells around the new formed bone trabeculae and large vessels in the defect in both groups (Figure S8, Supporting Information). More osteoclasts around the new bone trabeculae were observed in NGF@S/BMP‐2 group in comparation with BMP‐2@S group at 56 days post‐operation (**Figure** **8**A,B), suggesting that active bone remodeling in NGF@S/BMP‐2 group. We further evaluated the growth and distribution of TUBB3^+^ and CGRP^+^ nerve fibers in BMP‐2@S group and NGF@S/BMP‐2 group. Both sensory nerve markers including TUBB3 and CGRP were found to express higher in NGF@S/BMP‐2 group. (Figure 8A). In addition, we observed TUBB3 positive nerve fibers mainly travels around the trabecular space, which heavily overlapped with osteoclasts cell distribution.

Furthermore, we also validated this hypothesis using a Balb/c mouse subcutaneous transplantation model to avoid the complicated bone microenvironment and restrict the determining factors involved in this process. First, µCT showed bare scaffold implantation did not induce ectopic bone formation as expected, but BMP‐2 containing groups clearly formed mineralized tissue (Figure 8D). More specifically, quantitative µCT metrics of the mineralized tissue suggested that bone volume increment in NGF@S/BMP‐2 group was lower than BMP‐2@S group 28 days post‐implantation (Figure 8E–G). Correspondingly, neural immunofluorescence of TUBB3 and CGRP showed only NGF@S/BMP‐2 group was highly innervated with CGRP/TUBB3 positive nerve fibers. TUBB3 and CGRP nerve were hardly observed in control group and BMP‐2@S group, indicating that reduction in mineralized tissue formation might be related to higher level of innervation with engineered sensory nerve at the implantation sites.

These data demonstrated that engineered sensory nerve inhibited excessive ectopic bone formation when BMP‐2 was used as a morphogenetic cue. However, we also found that engineered sensory nerve was able to guide osteogenesis in both calvarial defect and subcutaneous models. Put together, engineered sensory demonstrated an adaptive response to different microenvironment: eSN tent to promote osteogenesis under bone defect scenario while it inhibits excessive bone formation when a robust osteogenic signal presents. In fact, although the adaptation of neural system in bone homeostasis has rarely been reported, neural adaption of organ homeostasis has been previously reported in both gut and vascular systems.^[^ ^24^ ^]^ Recently, accumulating evidences reported the adaptive role of sensory nerve in bone regeneration. These literature indicated that sensory nerve promoted ossification by promoting osteogenic differentiation of osteoblast,^[^ ^25^ ^]^ while sensory neve sense peripheral stimuli signaling to activate osteoclasts by regulating sympathetic nerves during orthodontics.^[^ ^26^, ^27^ ^]^ BMP‐2 is well known for its strong bone forming capability, as we as its side effects of causing excessive ectopic bone formation (Figures 7, 8). When sustained NGF supported eSN innervation in the presence of BMP‐2, the adaptation property of eSN proactively regulated osteogenesis by activating more robust bone remodeling process. In other words, when eSN is introduced in bone healing context, its self‐adaptation characteristics allow the healing process to adapt to its complicated microenvironment and physiological needs.

## 3 Conclusion

In summary, our study demonstrates that desired biomaterials for bone regeneration should couple the capacity of bone neurovascular network remodeling and rapid osteogenesis property. After reconstruction of sensory nerve after injury via NGF‐TrkA signaling pathway, it was found that neuralization and osteogenesis had synergistic effect. CGRP derived from sensory nerve accelerate the bone microvascular system regeneration and bone formation in vitro and in vivo. In addition, restoration of the sensory nerve microenvironment in the area of bone defect coordinated with BMP2 to optimize the osteogenesis process, avoid ectopic osteogenesis, and reduce the side effects of BMP‐2 in vivo. Therefore, this strategy provides an osteogenesis biomaterial through sensory nerve mediated neuromodulation, thus laying the foundation for achieving vascularized and neuralization osteogenesis, giving the bone with the “intelligence” to adapt to microenvironments, and providing an alternative therapeutic for large bone defect and fracture nonunion.

## 4 Experimental Section

All procedures involving animals were performed under the approval and guidance of Experimental Animal Ethics Committee of the Second Affiliated Hospital of Zhejiang University School of Medicine (Approval number: AIRB‐2021‐847).

### Materials

Decellularized matrix and beta‐tricalcium phosphate (*β*‐TCP) were provided by University of Shanghai for Science and Technology. Cell counting kit‐8 (CCK‐8) kit, alkaline phosphatase (ALP) kit, bicinchoninic acid (BCA) assay kit, and 4′,6‐diamidino‐2‐phenylindole (DAPI) were obtained from Beyotime Biotechnology Co (Shanghai, China). Phalloidin was obtained from Invitrogen Co. (USA). The Runx‐2, OCN, OPN, *β*‐actin antibodies were obtained from CST (MA, USA). Alexa Fluor 488‐labeled and Alexa Fluor588‐labeled Goat Anti‐Rabbit IgG (H+Lx) secondary antibodies were purchased from Jackson ImmunoResearch (West Grove, PA, USA).

### Preparation of Mineral Coated Microparticles (MCMs) in mSBF

The particles size of *β*‐TCP was almost 10 µm in diameter. To form the mineral coating on *β*‐TCP microparticles, the particles were incubated in modified simulated body fluid (mSBF) at 37 °C with 25 mm HCO_3_^−^ for 7 days with continuous rotation. As previous reports,^[^ ^28^ ^]^ the mSBF was prepared by adding the following reagents into deionized water in the order shown: 141 mm NaCl, 4 mm KCl, 0.5 mm MgSO_4_, 1.0 mm MgCl_2_, 25 mm NaHCO_3_, 20.0 mm HEPES, 5 mm CaCl_2_, and 2 mm KH_2_PO_4_. And the pH of mSBF should be adjusted to 6.80. Then, 25 mg *β*‐TCP particles were incubated in 50 mL mSBF in a conical tube to form the mineral coatings. And the mSBFs were refreshed daily throughout the entire coating process in order to maintain consistent ion concentration for mineral coating growth. After 7 days of incubation, the MCMs were rinsed with deionized water and lyophilized.

### Preparation of ECM@S and NGF@S

ECM@S was prepared by porcine acellular dermal matrix. In brief, the porcine skin was cut into 8 cm × 8 cm × 0.5 cm sections, and cleaned with deionized water. Then, the epidermis and subcutaneous tissue were removed to obtain 1.5 mm thickness dermis designated. Thereafter, the pieces of the dermis were then purified using the ternary solution for 6 h at 75 °C to remove fat tissue and cells. Carefully washed dermis samples were immersed in 40 U mL^−1^ DNase I solution with 10 mm MgCl_2_ for 90 min at 37 °C (170 rpm) to completely remove cell debris. Finally, DNase I‐treated samples were then washed with PBS for 90 min and then sterilized with a solution of 0.1% peracetic acid/4% ethanol for 2 h to preserve decellularized matrix (dECM). The decellularized matrix (dECM) were rinsed with PBS buffer for several times and the pH of decellularized matrix had to be adjusted to 7.20. 5 µL NGF (500 ng) were added to prepared decellularized matrix and fully mixed. The mixture was then placed in a mold, frozen at −80 °C and freeze‐dried to obtain the ECM@S and NGF@S.

### Binding and Release of BMP‐2/NGF from NGF@S/BMP‐2

MCM‐BMP‐2 particles were prepared by binding BMP‐2 into MCM microparticles. In brief, 5.0 mg of each type of MCMs was incubated in 1.0 mL solutions with 1200 ng mL^−1^ BMP‐2 in 0.2% BSA solution at 37 °C for 4.0 h with rotation. The MCM‐BMP‐2 particles were then centrifuged at 12 000 RPM for 2 min and washed with 1.0 mL PBS twice. Particularly, BMP‐2 concentration in the supernatant and washed PBS were evaluated by BMP‐2 Elisa assay for calculating binding efficiency. For dECM‐MCM scaffold, the decellularized matrix (dECM) was rinsed with PBS buffer for several times and the pH of decellularized matrix had to be adjusted to 7.20. 108 mg MCM particles or MCM‐BMP‐2 particles were mixed with 2.242 mL decellularized matrix (dry weight: 1.7936 mg), and then placed the mixture in a mold and freeze at −80 °C before lyophilizing to obtain ECM‐MCM@S or BMP‐2@S. Prepared scaffold was stored at 20 °C for further evaluation. For binding NGF to BMP‐2@S, 5 µL NGF (500 ng) were added to lyophilized scaffold to obtain NGF@S/BMP‐2. For releasing of BMP2 and NGF from NGF@S/BMP‐2, NGF@S/BMP‐2 (0.2 mg) from lyophilization were incubated in 1.0 mL Tris buffered saline (TBS, pH 7.40) at 37 °C and the released protein amount was determined by BMP‐2 Elisa assay and NGF Elisa assay.

### Characteristic of MCM Particles and Scaffolds

The morphology and composition of the MCMs and scaffolds were examined by scanning electron microscopy (SEM), Fourier transform infrared (FTIR) spectroscopy, and X‐ray diffraction (XRD). The surface morphology of the MCMs and scaffold were observed by SU‐8010 field emission scanning microscopy (FESEM, Hitachi, Japan) after sputter‐coating with gold. FTIR was employed to analyze the composition of the mineral coating on MCMs using an Equinox 55 spectrometer (Bruker AXS, Germany). The FTIR spectra of the samples were recorded from 400 to 2000 cm^−1^ in transmission mode using potassium bromide pellets. The phase composition of the mineral coating was identified by XRD with a Hi‐Star X‐ray diffractometer with CuK*α* radiation.

### Calcium Released from Scaffold

As previous reports, 5.0 mg of the ECM scaffold was incubated in 1.0 mL TBS in a 1.5 mL Eppendorf tube at 37 °C. The releasing buffer was collected from the tube at various predetermined time points and 1.0 mL fresh TBS was refilled into the tube. The calcium amount released from the scaffold was quantified by an Arsenazo III based assay. Briefly, 5 µL of sample was mixed with 195 µL of 0.4 mm Arsenazo III in 20 mm Tris buffer (pH 7.40). The amount of calcium was then detected by measuring absorbance at 615 nm using a Molecular Device microplate reader, and the concentration was calculated by a set of standards with predetermined calcium concentrations.

### Cell Culture

Schwann cells, RAOECs, rat BMSCs and Human BMSCs were obtained from the commercial approach and used in vitro experiments, which were incubated in Dulbecco's Modified Eagle Media (DMEM) (Gibco, USA) supplemented with 10% fetal bovine serum and 100 µg mL^−1^ penicillin/streptomycin in 5% CO_2_ at 37 °C. Osteoblastic differentiation of MSCs was carried out using osteoblastic induction medium (OIC) containing standard growth medium supplemented with 10^−8^ m dexamethasone, 50 µg mL^−1^ ascorbic acid, and 10 mm *β*‐glycerophosphate (Sigma‐Aldrich, USA).

### Reverse Transcription and Real Time PCR

To interrogate osteogenic and neurogenic gene expression, samples were collected in Trizol Reagent (Invitrogen) for PCR analysis following the manufacturer's instructions. After RNA isolation, 1000 ng of RNA was reverse‐transcribed with the QuantiTect Reverse Transcription Kit (QIAGEN, Valencia, CA), and qPCR was performed using the QuantiFast Probe PCR Kit (QIAGEN) on a QuantStudio 5 system (Applied Biosystems). Primers and probes for housekeeping genes GAPDH, RUNX2, COL1AL, OSX and ALP were synthesized by Qingke biotech Co., Ltd. (Table S1). Amplification conditions were 95 °C for 5 min, followed by 45 cycles at 95 °C for 10 s and 60 °C for 30 s. qPCR results were normalized to GAPDH transcript levels to yield △*Ct* and cells from fresh BMA to yield △△*Ct*. Last, fold change in expression relative to the untreated and housekeeping gene was calculated using 2^−△△^ *^Ct^*.

### Cell Culture in 3D Scaffold

First, prepared scaffolds were washed with PBS for three times and then cells (20 × 10^4^ cells per Scaffold) were seeded on different scaffolds (ECM@S, ECM‐MCM@S, NGF@S, BMP‐2@S, NGF@S/BMP‐2). Once the cells were attached to the scaffolds, cell‐seeded scaffolds were transferred to new wells containing culture medium and cultured in a humidified incubator at 37 °C with 5% CO_2_.

### Biocompatibility Test

BMSCs (20 × 10^4^ per scaffold) were seeded in prepared scaffolds (Radius: 3 mm) and cultured with DMEM containing 10% FBS. After 1, 3, and 7 days of incubation, CCK‐8 reagent (ck04, Dojindo, Japan) was mixed with fresh DMEM medium for culturing 2 h and cell viability was quantified by a microplate reader at 450 nm (TECAN, Switzerland). Additionally, Live/Dead kit (Invitrogen, US) was comprised of Calcein‐AM (green fluorescence) and ethidium homodimer‐1 (red fluorescence) that was employed to assess BMSCs viability. And the fluorescent images were acquired by a laser scanning confocal microscope (LSCM, Zeiss, Germany).

### Rat Calvarial Defect Model

Rats underwent bilateral calvarial osteotomies as previously described,^[^ ^29^ ^]^ and treatment of experimental animals was in accordance with the guidance of the Animal Care and Use Committee of Medical College of Zhejiang University and all National Institutes of Health animal handling procedures. The animals had free access to both sterile water and food in a light and temperature‐controlled environment. All experimental rats were used between 8 and 10 weeks of age and had an average weight of 200 ± 10 g. SD Rats were anesthetized, and bilateral, critical sized, full‐thickness bone defect with 6 mm in diameter was trephined in parietal bone. Briefly, 8‐week‐old rats were anesthetized with an intraperitoneal injection of 4% pentobarbital sodium (40 mg kg^−1^). The head of rats was skinned and the surgical site was cleaned with iodine. Then, an incision was made just off the sagittal midline to expose the parietal bone. The pericranium was removed, and 6 mm defects were made on one side of non‐suture‐associated parietal bone using a trephine drill, avoiding perforation of the dura. The surgical area was cleaned with saline and each defect was filled with prepared ECM scaffold (Each defect was implanted with ECM@S, MCM‐MCM@S, BMP‐2@S, NGF@S, NGF@S/BMP‐2, respectively.) (Radius: 3 mm). After implantation, the skin was sutured. Rats were sacrificed by an intraperitoneal injection of over‐dose pentobarbital sodium at 15 days, 28 days, and 58 days post‐operation, then the skull were acquired and stored in 4% paraformaldehyde for µCT evaluation and histological analysis.

### Rats’ Bone Evaluation by µCT

After 4 or 8 weeks, calvarial tissues were harvested, fixed in 4% formaldehyde solution for 48 h, and scanned with a µCT scanner (Bruker SkyScan 1172, Kontich, Belgium). Scanning was performed with a resolution of 10 µm, an exposure time of 190 ms, and 475 projections were acquired at the angle of 190°. µCT images were acquired with 0.5 mm Al filtration at 104 kV and 98 mA. During scanning the samples were wrapped in paper soaked in PBS to avoid dehydration. The relative of new bone was evaluated using Image J, and BV/TV were analyzed using the SkyScan CT‐Analyzer program (Bruker micro‐CT).

### Histological Analysis

Before histological staining, all fixed tissues were treated with 10% EDTA solution for 20 days for decalcification. Then prepared tissues were dehydrated and embedded in paraffin. After cut at a thickness of 5 µm, sections were stained with H&E and Masson trichrome, respectively. Then sections were observed using a microscope to evaluate new bone formation.

### Immunofluorescence

Slides of each tissue were dewaxed and dehydrated with gradient alcohol. The antigenic repair was performed by trypsin method, and then rinsed with PBS for three times. Then the sections were permeabilized with 0.5% v/v Triton X‐100 for 20 min, and blocked with 1% w/v goat serum albumin for 1 h at 37 °C. The samples were then probed at 4 °C overnight with antibodies against TUBB3, NGF, RUNX‐2, OCN, and incubated for 1 h with a secondary antibody. Finally, nuclei were stained for 5 min with 0.1 g mL^−1^ 4′,6‐diamidino‐2‐phenylindole (DAPI), and samples were washed and imaged by Leica fluorescence microscope.

### Statistical Analysis

The data were evaluated by one‐way analysis of variance (ANOVA) followed by post hoc Tukey's multiple comparison test. The data were presented as mean ± standard deviation (*n* ≥ 3, independent samples) and a difference of *p < 0.05 was considered significant.

## Conflict of Interest

The authors declare no conflict of interest.

## Author Contributions

Z.Z.: Conceptualization, Writing—original draft, Writing—review & editing; F.W.: Conceptualization, Writing—original draft, Writing—review & editing; X.H.: Conceptualization, Writing—original draft, Writing—review & editing; H.S.: Methodology; J.X.: Methodology; H.Q.: Methodology; X.Yan: Investigation; W.S.: Investigation; W.T.: Investigation; X.J.: Visualization; Z.S.: Visualization; Y.Z.: Visualization; S.Z.: Visualization; Y.W.: Supervision, Writing—review & editing; X.Yu: Supervision, Writing—review & editing; Z.Y.: Supervision, Writing—review & editing.

## Supporting information

Supporting Information

Supporting Information

## Data Availability

All data are available in the main text or the supplementary materials.
