# Supplementary material for: Engineered Sensory Nerve Guides Self‐Adaptive Bone Healing via NGF‐TrkA Signaling Pathway
Source: Adv Sci (Weinh). 2023 Feb 1;10(10):2206155. doi: 10.1002/advs.202206155 (PMC10074090; doi:10.1002/advs.202206155)
Supplement: Supplementary file 1 — Supporting Information [file ADVS-10-2206155-s001.pdf]

ADVANCED SCIENCE
Open Access

Supporting Information

for *Adv. Sci.*, DOI 10.1002/advs.202206155

Engineered Sensory Nerve Guides Self-Adaptive Bone Healing via NGF-TrkA Signaling Pathway

Zengjie Zhang, Fangqian Wang, Xin Huang, Hangxiang Sun, Jianxiang Xu, Hao Qu, Xiaobo Yan, Wei Shi, Wangsiyuan Teng, Xiaoqiang Jin, Zhenxuan Shao, Yongxing Zhang, Shenzhi Zhao, Yan Wu, Zhaoming Ye* and Xiaohua Yu**

FRONT MATTER

Engineered Sensory Nerve Guides Self-Adaptive Bone Healing via NGF-TrkA Signaling Pathway

Zengjie Zhang[1,2,3]*, Fangqian Wang[1,2,3]*, Xin Huang[1,2,3]*, Hangxiang Sun[1,2,3], Jianxiang Xu[1,2,3], Hao Qu[1,2,3], Xiaobo Yan[1,2,3], Wei Shi[3,4], Wangsiyuan Teng[1,2,3], Xiaoqiang Jin[1,2,3], Zhenxuan Shao[1,2,3], Yongxing Zhang[1,2,3], Shenzhi Zhao[1,2,3], Yan Wu[1,2,3]†, Xiaohua Yu[1,2,3]†, Zhaoming Ye[1,2,3]†

1. Department of Orthopedic Surgery, the Second Affiliated Hospital, Zhejiang University School of Medicine, Hangzhou City, Zhejiang Province, PR China

2. Orthopedics Research Institute of Zhejiang University, Hangzhou City, Zhejiang Province, PR China

3. Key Laboratory of Motor System Disease Research and Precision Therapy of Zhejiang Province, Hangzhou City, Zhejiang Province, PR China

4. Department of Orthopedic, Taizhou First People's Hospital, Wenzhou Medical University, Taizhou City, Zhejiang Province, PR China

* Equally contributed to this work

† Corresponding author

Correspondence to: Zhaoming Ye, Xiaohua Yu or Yan Wu, Department of Orthopaedics, The Second Affiliated Hospital of Zhejiang University School of Medicine, Hangzhou 310009, China

E-mail address: yezhaoming@zju.edu.cn (Zhaoming Ye), xiaohua.yu@zju.edu.cn (Xiaohua Yu), wuyanzju@zju.edu.cn (Yan Wu)

† Three authors contribute equally.

Keywords: Extracellular matrix, Nerve growth factor, Sensory nerve, BMP-2, Osteogenesis

Supplementary Materials

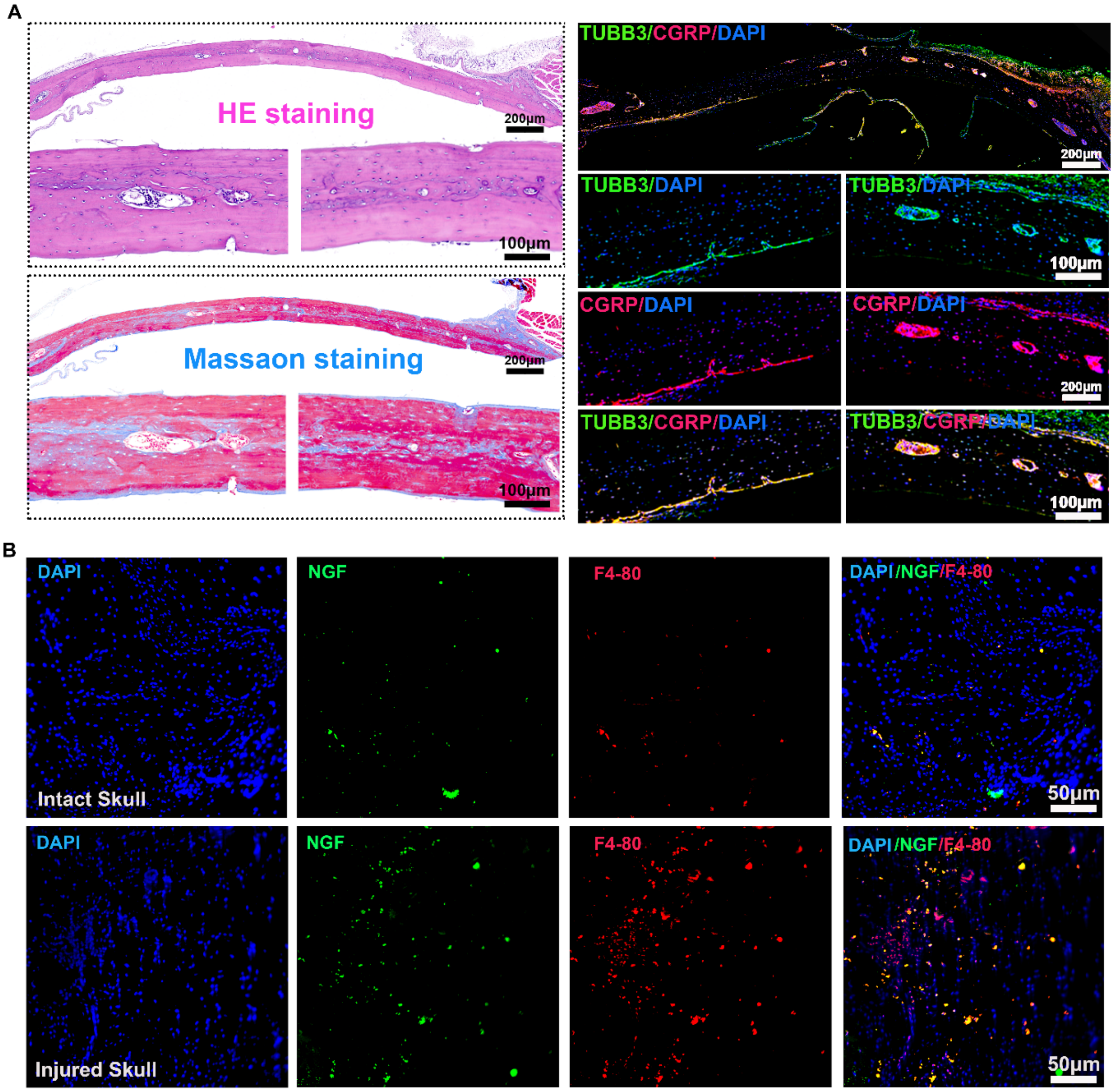

Supplementary Figure1. (A) Representative HE and Masson Staining of uninjured cranial bone and representative fluorescence images showing TUBB3+ nerve fibers (Green) and CGRP+ nerve fibers (Red) in the uninjured cranial bone. (B) Representative NGF and F4-80 immunofluorescent of intact and injured cranial bone after operation. n=3 animals with per time point. Data are represented as mean ± SD.

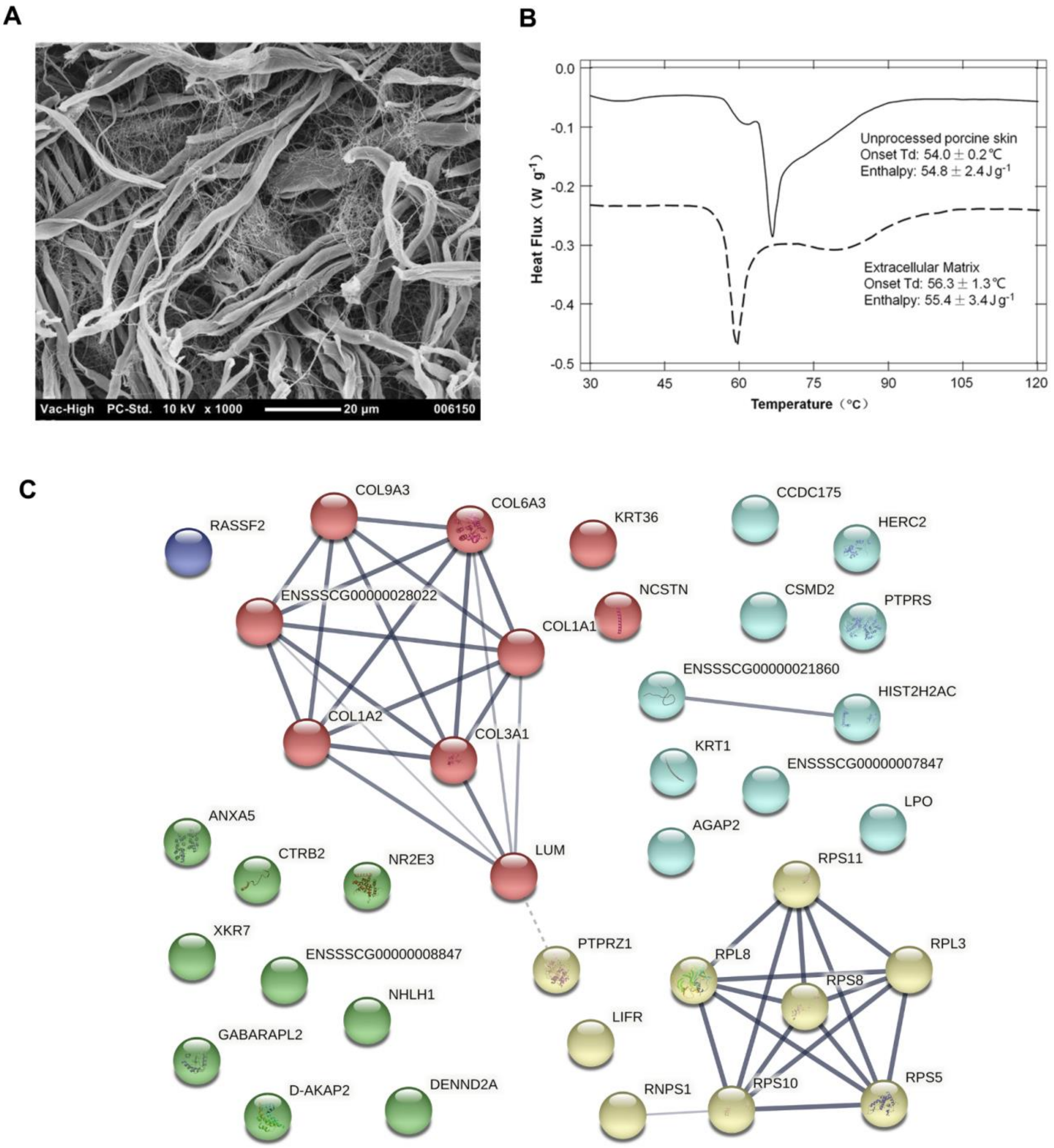

Supplementary Figure2. (A) Representative SEM image of extracellular matrix fibers (Bar: 20μm). （B）Heat flux of unprocessed porcine skin and extracellular matrix. (C) Kmeans Clusting cluster analysis: Several extracellular matrix collagen and egg adhesion (pink) accounted for 92.04%, ② cytoskeletal proteins (lower left green) accounted for 0.29%, ③ cytoplasmic proteins (upper right blue) accounted for 0.60%, ④ nuclear proteins (lower right light yellow) accounted for 0.10%.

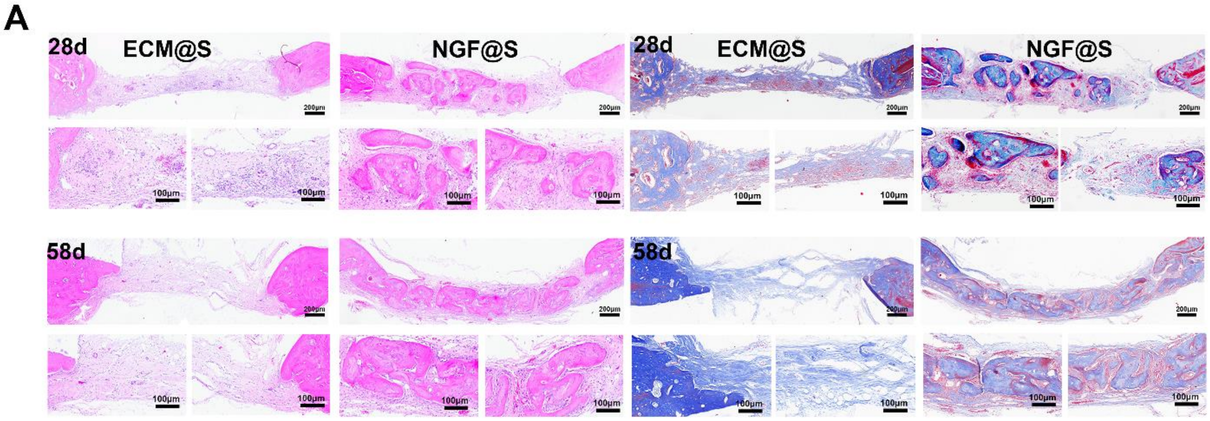

Supplementary Figure3. (A) HE-stained and Masson-stained images of cranial defect in different groups at 28days or 56days after implantation. n=3 animals with 6 bone defects per time point and per group. Data are represented as mean ± SD.

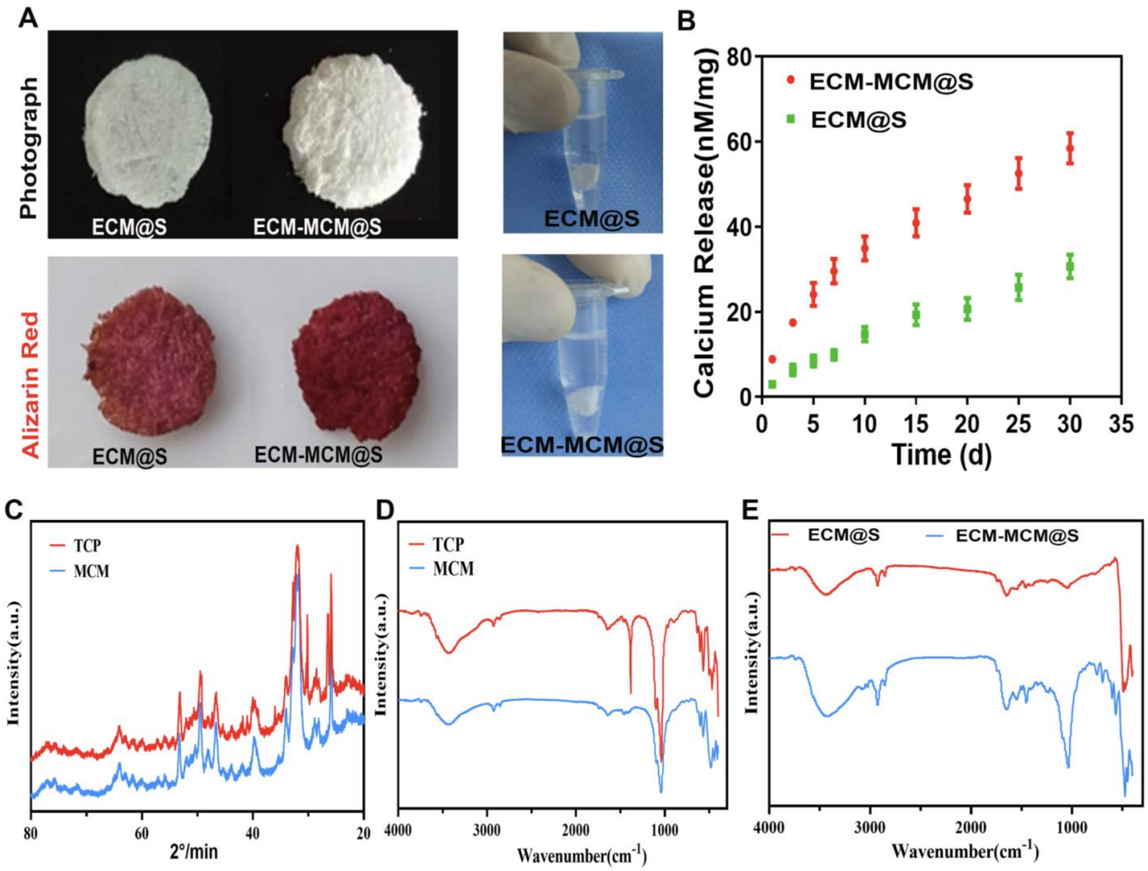

Supplementary Figure4. (A) Representative images of ECM@S and ECM-MCM@S, ARS staining of each scaffold and morphological change of scaffold in distilled water. (B) Calcium release curve of different scaffold in distilled water. (C, D, E) Fourier infrared spectrometer of β-TCP, mineral coated microparticles（MCM）ECM@S and ECM-MCM@S.

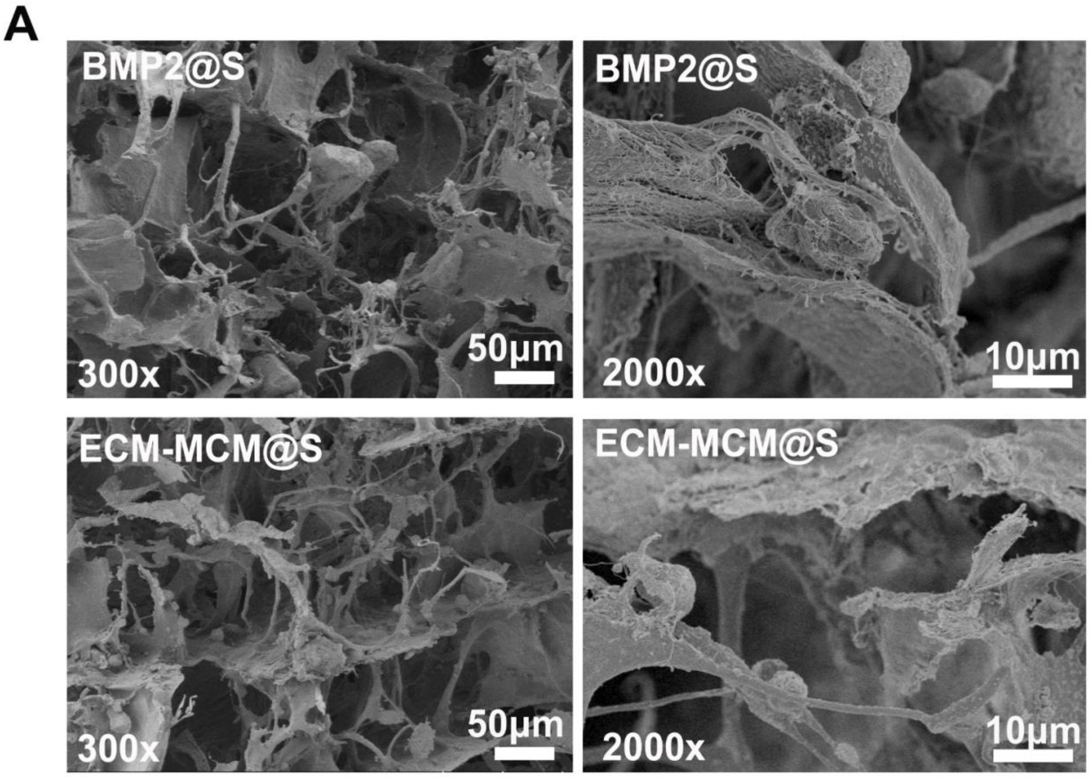

Supplementary Figure5. (A) Representative SEM images of BMP@S and ECM-MCM@S （Bar：50μm and 10μm）

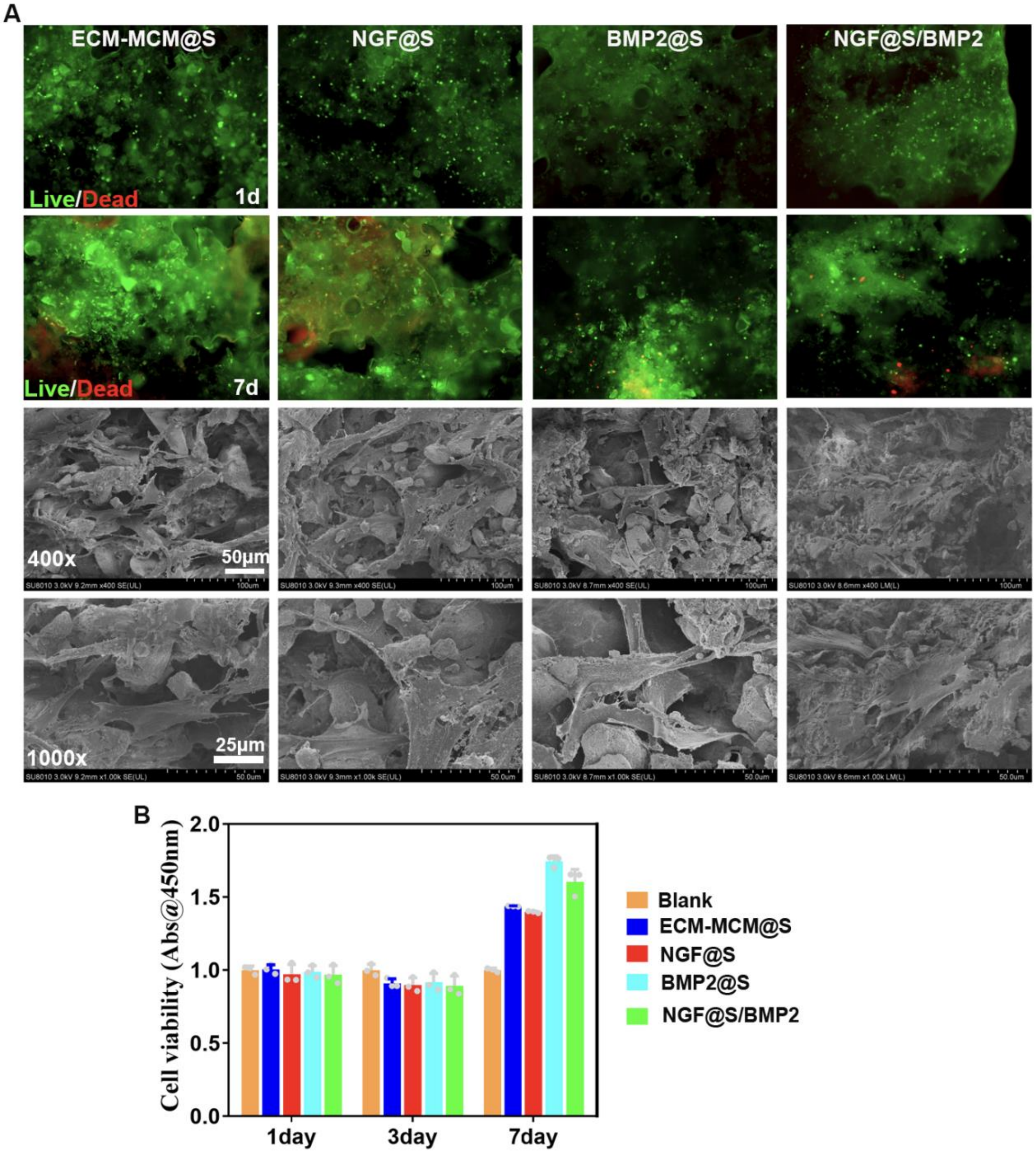

Supplementary Figure6. (A) Representative images of live and dead staining of BMSCs in different modified scaffolds (live：Green; dead: Red), and SEM images of BMSCs in different scaffolds. (B) The toxicity of each scaffold was evaluated by cck8 kit. n=3 times per experiments. Data are represented as mean ± SD.

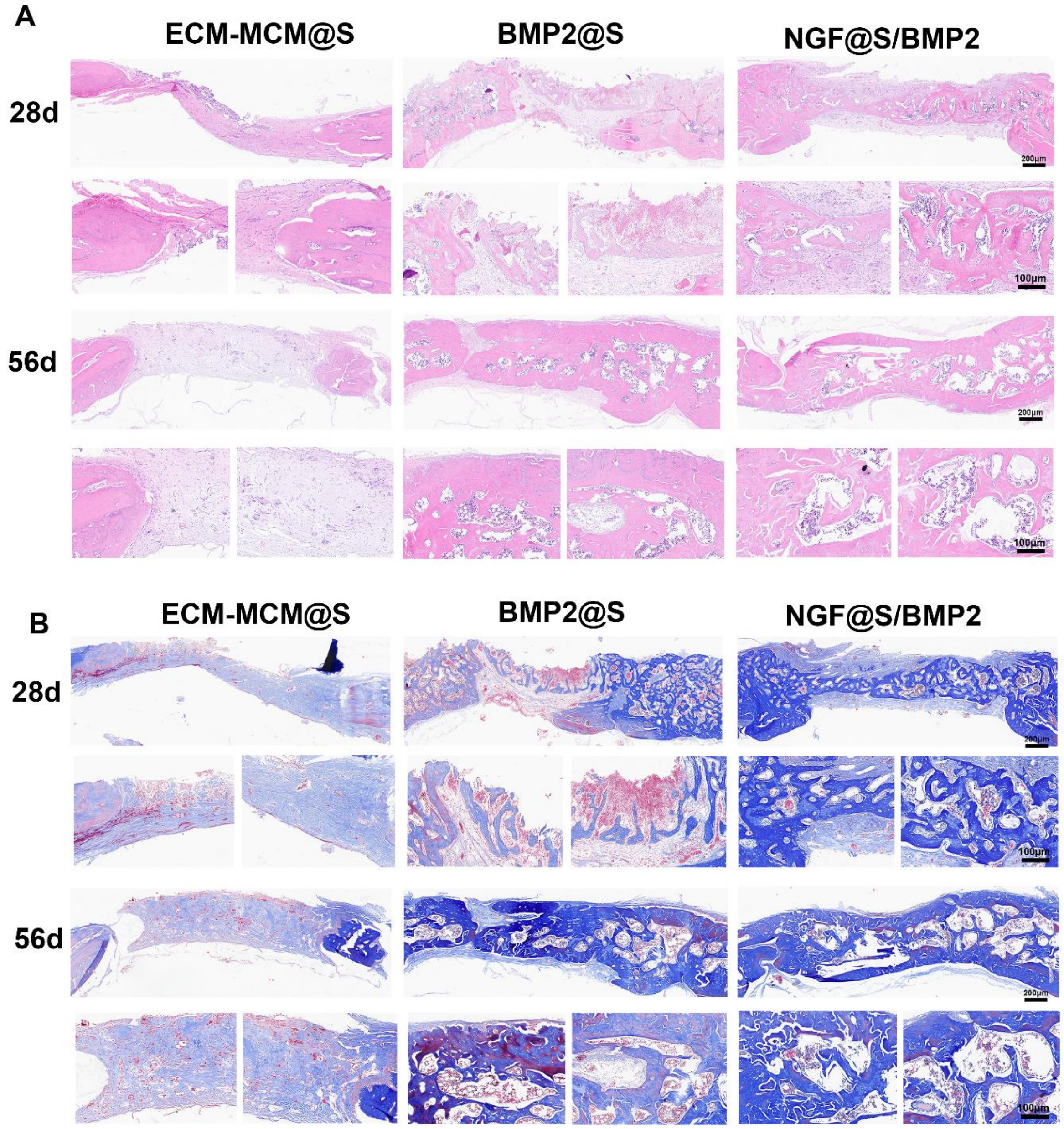

Supplementary Figure7. (A) HE-stained and Masson-stained images of cranial defect in different groups at 28days or 56days after implantation. n=3 animals with 6 bone defects per time point and per group. Data are represented as mean ± SD.

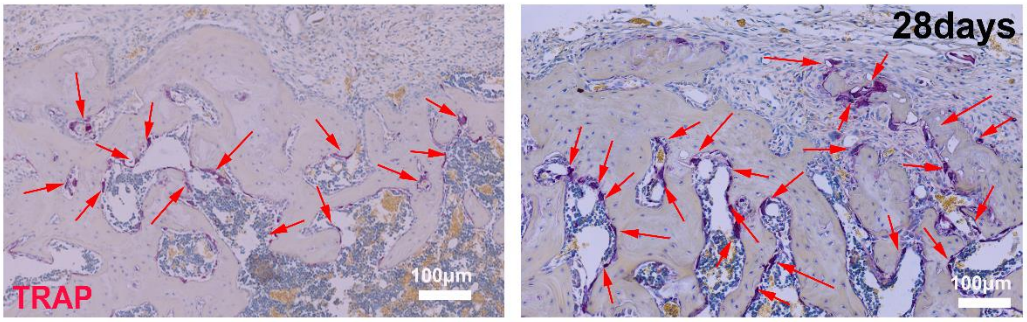

Supplementary Figure8. (A) Representative image of Trap staining of cranial bone in different groups and quantification of Trap positive cells in each group. n=3 animals with 6 bone defects per time point and per group. Data are represented as mean ± SD.
