# Supplementary material for: Engineered Sensory Nerve Guides Self‐Adaptive Bone Healing via NGF‐TrkA Signaling Pathway
Source: Adv Sci (Weinh). 2023 Feb 1;10(10):2206155. doi: 10.1002/advs.202206155 (PMC10074090; doi:10.1002/advs.202206155)
Supplement: Supplementary file 2 — Supporting Information [file ADVS-10-2206155-s002.pdf]

ADVANCED SCIENCE
Open Access

Supporting Information

for *Adv. Sci.*, DOI 10.1002/advs.202206155

Engineered Sensory Nerve Guides Self-Adaptive Bone Healing via NGF-TrkA Signaling Pathway

Zengjie Zhang, Fangqian Wang, Xin Huang, Hangxiang Sun, Jianxiang Xu, Hao Qu, Xiaobo Yan, Wei Shi, Wangsiyuan Teng, Xiaoqiang Jin, Zhenxuan Shao, Yongxing Zhang, Shenzhi Zhao, Yan Wu, Zhaoming Ye* and Xiaohua Yu**

Table S1

Primer name	Primer sequence (5′to3′)
Runx2（F)	CACTGGCGCTGCAACAAGA
Runx2 (R)	CATTCCGGAGCTCAGCAGAATAA
ALP（F)	CCTTGTAGCCAGGCCCATTG
ALP（R)	GGACCATTCCCACGTCTTCAC
GAPDH（F)	GCACCGTCAAGGCTGAGAAC
GAPDH（R)	TGGTGAAGACGCCAGTGGA
COL1A1（F)	TCTAGACATGTTCAGCTTTGTGGAC
COL1A1（R)	TCTGTACGCAGGTGATTGGTG
OSX (F)	GCCAGAAGCTGTGAAACCTC
OSX (R)	GCT GCAAGCTCTCCATAA CC
